# Determination of Stiffness and the Elastic Modulus of 3D-Printed Micropillars with Atomic Force Microscopy–Force Spectroscopy

**DOI:** 10.1021/acsami.2c21921

**Published:** 2023-01-27

**Authors:** Giorgio Cortelli, Leroy Grob, Luca Patruno, Tobias Cramer, Dirk Mayer, Beatrice Fraboni, Bernhard Wolfrum, Stefano de Miranda

**Affiliations:** † Department of Civil, Chemical, Environmental and Materials Engineering, University of Bologna, Viale del Risorgimento 2, 40136 Bologna, Italy; ‡ Department of Physics and Astronomy, University of Bologna, Viale Berti Pichat 6/2, 40127 Bologna, Italy; § Neuroelectronics, Munich Institute of Biomedical Engineering, Department of Electrical Engineering, Technical University of Munich, 85748 Garching, Germany; ∥ Institute of Biological Information Processing (IBI-3), Forschungszentrum Jülich GmbH, 52425 Jülich, Germany

**Keywords:** atomic force microscopy, micromechanics, micropillar, inkjet printing, additive manufacturing

## Abstract

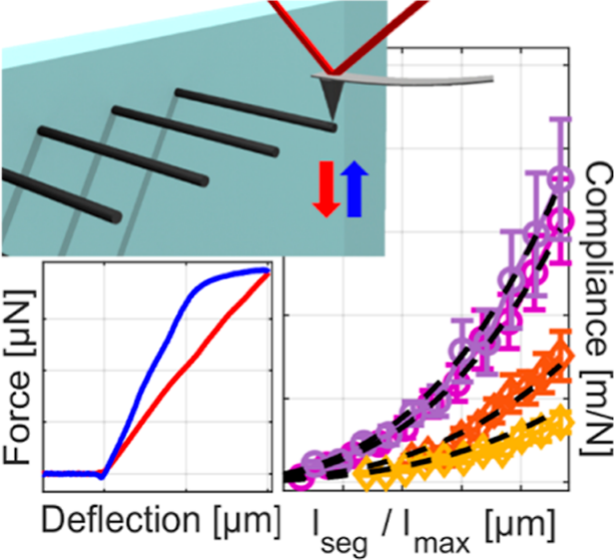

Nowadays, many applications in diverse fields are taking advantage of micropillars such as optics, tribology, biology, and biomedical engineering. Among them, one of the most attractive is three-dimensional microelectrode arrays for in vivo and in vitro studies, such as cellular recording, biosensors, and drug delivery. Depending on the application, the micropillar’s optimal mechanical response ranges from soft to stiff. For long-term implantable devices, a mechanical mismatch between the micropillars and the biological tissue must be avoided. For drug delivery patches, micropillars must penetrate the skin without breaking or bending. The accurate mechanical characterization of the micropillar is pivotal in the fabrication and optimization of such devices, as it determines whether the device will fail or not. In this work, we demonstrate an experimental method based only on atomic force microscopy–force spectroscopy that allows us to measure the stiffness of a micropillar and the elastic modulus of its constituent material. We test our method with four different types of 3D inkjet-printed micropillars: silver micropillars sintered at 100 and 150 °C and polyacrylate microstructures with and without a metallic coating. The estimated elastic moduli are found to be comparable with the corresponding bulk values. Furthermore, our findings show that neither the sintering temperature nor the presence of a thin metal coating plays a major role in defining the mechanical properties of the micropillar.

## 1 Introduction

Micropillars are three-dimensional microstructures characterized by a very large extension in one dimension, resulting in a great aspect ratio. Nowadays, many applications in diverse fields are taking advantage of micropillars such as optics, tribology, biology, and biomedical engineering.^1−4^ Usually, micropillars are fabricated in a cleanroom by subtractive processes such as photolithography in combination with dry and wet etching, wire-electrode cutting, bulk micromachining, and laser cutting.^5−8^ One microfabrication method that is gaining increased popularity is 3D inkjet printing. It is an attractive technique for micropillar production because it allows flexible, room-temperature, scalable, and economical fabrication processes.^9−11^ Among different applications of micropillars in biomedical engineering, perhaps the most relevant are 3D microelectrode arrays (3D MEAs).

Three-dimensional microelectrode arrays have attracted considerable interest due to their use in various applications for in vivo and in vitro studies, such as cellular recording, biosensors, and drug delivery.^1−12^ This is because three-dimensional structures allow for increased device area, spatial resolution, and signal-to-noise ratio. In the case of cellular recording, conductive micropillars are used as the interface between the device and the cell under investigation to measure action potentials.^13−22^ Regarding biosensors and biomedical implants, the micropillar-based electrodes act as a vital component for monitoring organ activity and electrically/optically/thermally stimulated therapy.^23−28^ In the electrode–tissue interface, the mechanical mismatch between the tissues and the electrode should be minimized to avoid invasive tissue damage and related loss of devices. Damaged tissue and scar formation ultimately cause weakly coupled electrode–tissue interfaces with strong attenuation of recorded signals. In addition, implantable microelectrodes have to be designed for long-term applications, so they must adapt to the mechanical strains exerted by the surrounding tissue while maintaining good coupling with the tissue for recording and stimulation.^29−33^ Pillar-like structures, called microneedles, are often used in drug delivery patches to penetrate the skin and release the drug.^34^ The drug is loaded into the pores of the microneedle and can diffuse out of the pores when the matrix penetrates the skin. In this case, microneedles must be able to penetrate the human stratum corneum (∼10–20 μm) without breaking or bending. Needle breakage or failure during or after patch application may alter the drug release profile, leading to premature and uncontrolled drug release.

Accurate characterization of the mechanical properties of individual micropillars is a very challenging but crucial aspect in the fabrication and optimization of patterned surfaces and biomedical devices in different applications. In fact, the mechanical properties of individual micropillars vary depending on the material, geometry, and fabrication method and are critical in determining whether the device will fail or perform properly. The characterization of the mechanical properties of individual micropillars is limited by the lack of experimental techniques that are easy to access and use. Most characterization methods available nowadays require that the instrument used for mechanical testing be mounted inside a scanning electron microscope. The use of a scanning electron microscope is necessary to monitor the displacement in real-time during the mechanical test. Nanoindenters, tensile machines, or atomic force microscopes inside a scanning electron microscopy (SEM) chamber have been used to test tensile, compression, and bending of micropillars.^35−44^ Although these approaches lead to reliable results, they require expensive and difficult-to-use instrumentation. For these reasons, some early experimental techniques with AFM that do not involve SEM have been proposed. These techniques consist of bending tests on pillar-like structures clamped at both ends or cantilevered.^45−47^ However, most of these methods have been developed for samples obtained using top-down fabrication techniques and require specific designs to hold the sample. It is therefore crucial to develop techniques that allow us to measure the mechanical properties of individual micropillars, considering their actual geometry. This is indeed a crucial step for design and optimization, so it is desirable to develop techniques for rapid implementation, which require only easy-to-access equipment.

In this paper, we demonstrate a reliable and easy-to-operate atomic force microscopy (AFM) method to characterize the stiffness and elastic modulus of inkjet-printed micropillars. The method is based on force spectroscopies performed with the AFM tip in contact with the sidewall of the micropillar. By measuring the deflection of the micropillar at different lengths, we obtained the stiffness as a function of the micropillar length. In the measurements, we find that the tangential force acting on the AFM tip has a significant impact, causing an apparent stiffness variation between loading and unloading. To analyze the data, we introduce a mechanical model that relates the stiffness variation with the length to the micropillar geometry and elastic material properties. To test our method, we characterized micropillars obtained using different inks and microfabrication procedures. Sintered, porous, metal nanoparticle-based pillars were compared to similarly sized polymeric structures. Specifically, four types of samples were considered: silver nanoparticle-based micropillars sintered at 100 and 150 °C and polyacrylate (PA) micropillars with and without a metallic coating. Silver, commonly used as a conductive material in inkjet printing, was sintered at lower temperatures to promote its use on thermally sensitive substrates. PA was chosen as a non-porous equivalent to the silver-based micropillar (view Figure S1 in the Supporting Information for internal structures of both micropillar types). We decided to test Ag micropillars, as they have already been used for in vitro applications.^9^ We then tested PA micropillars, as the material has a significantly lower elastic modulus (by almost 2 orders of magnitude), confirming that the experimental method can also be used for less rigid materials. Our measurements highlight that all the fabricated micropillars show a stiffness—pillar length relation that follows a cubic power-law. Accordingly, the printed micropillar can be modeled as a beam with a circular cross-section of constant radius.^48^ The elastic moduli obtained for the pillars are consistent with those for the corresponding bulk materials, and only minor variations are found for pillars obtained with lower sintering temperatures or for pillars with metal coating.

## 2 Results and Discussion

**Figure 1:**
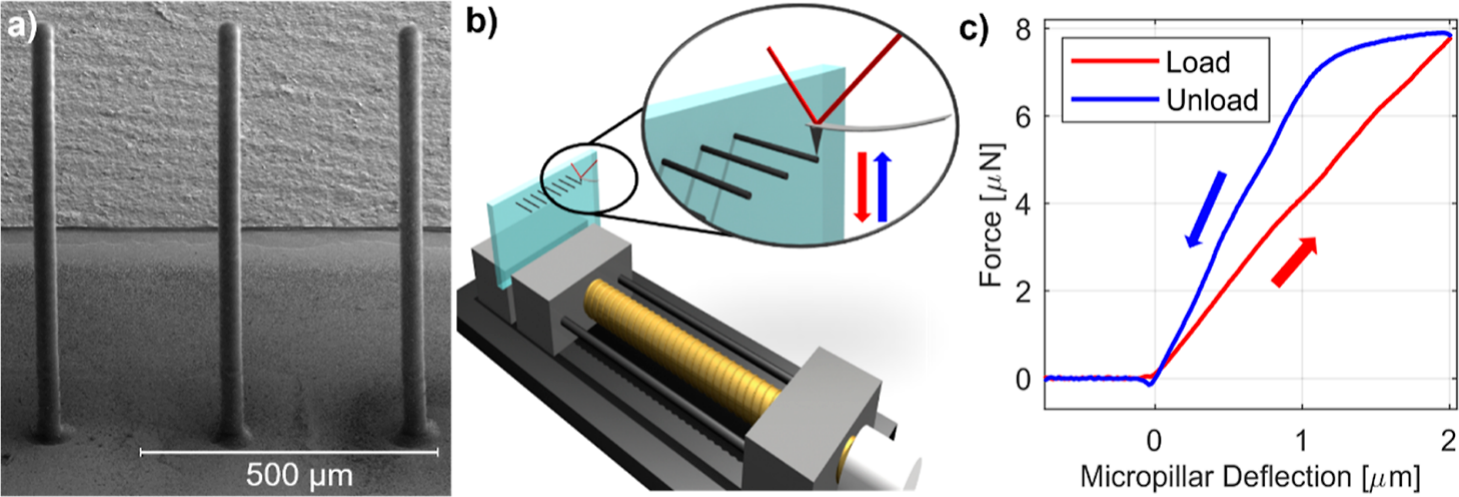
Sample and setup. (a) SEM image of PA micropillars. (b) Scheme of the sample holder and the samples under the AFM tip. (c) Typical force–micropillar deflection curve acquired with the setup. The red and blue arrows indicate the loading and unloading curves, respectively.

Our experimental method is based on force spectroscopy, in which the AFM tip exerts a cyclic load applied to the sidewall of the 3D inkjet-printed micropillar (Figure 1a). A scheme of the experimental setup is shown in Figure 1b. Initially, the contact between the AFM tip and the sample is established. Then, the probe is moved, following a trajectory along the *Z* axis until a maximum user-defined displacement is reached. Afterward, the direction of displacement is reversed to conclude a measurement cycle. To study bending, it is necessary to apply the load to the lateral surface of the micropillar. Therefore, a dedicated sample holder was designed to position the micropillar in the *XY* plane of the measurement system. By moving in the *Z* direction, the AFM tip can approach and bend the micropillar. A representation of the micropillar under the AFM tip is shown in the inset of Figure 1b. The experimental setup requires that the samples fulfill only two specific requirements: (i) the stiffness of the substrate must be sufficiently high to avoid rotations at the base, and (ii) the position of the micropillars on the substrate must be relatively close to edges to avoid blocking of the AFM laser before reaching the position sensitive photodetector (PSPD). In order to meet (i), we glued the final 3D-printed micropillar array to glass slides. To meet (ii), we microfabricated 3D micropillars such that they were close to the edge of the polyethylene naphthalate (PEN) substrate. It should be noticed that the experimental method is versatile and can be applied to very different samples, as the requirements are not particularly stringent. Similar techniques are reported in^46^ and,^47^ although in that case, nanostructures were targeted. A detailed description of the sample treatment for bonding the slide to the substrate and the fabrication of micropillars close to the substrate edge can be found in the Experimental Section/Methods.

**Figure 2:**
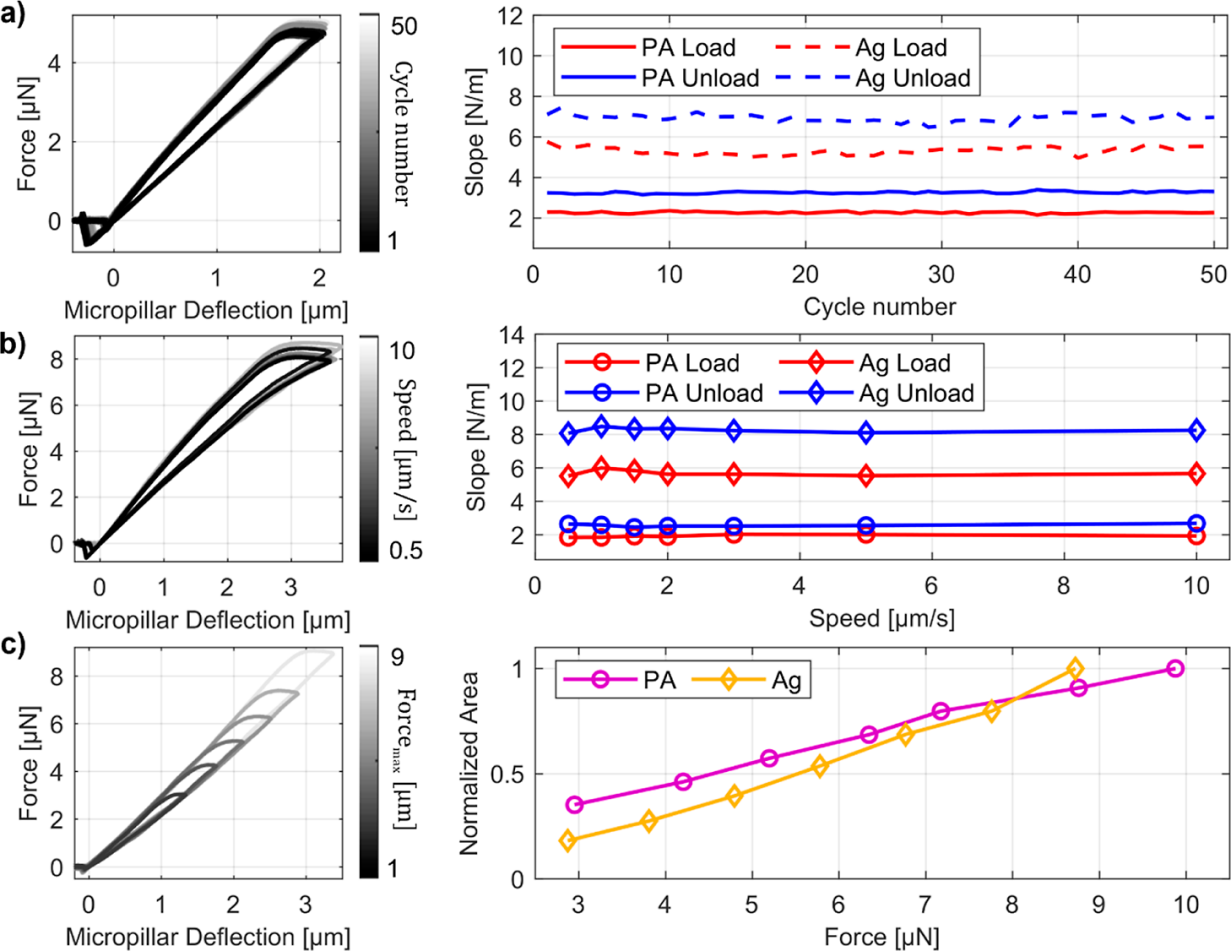
Investigation of hysteresis in force–deflection curves measured on PA and Ag micropillars. (a) Force–deflection curves and extracted slopes of loading and unloading for repeated measurement cycles. (b) Force–deflection curves and extracted slopes of loading and unloading acquired at different loading speeds. (c) Force–deflection curves and extracted hysteresis area as a function of the maximum loading force reached.

The deflection of the micropillar is determined by subtracting the deflection of the AFM cantilever from the total displacement measured along the *Z* axis. This is valid as long as the indentation of the AFM tip into the microelectrode surface is negligible compared to the deflection of the microelectrode itself (see Figure S2). A force–deflection curve obtained with our experimental setup on a metal-coated PA micropillar is shown in Figure 1c. Both the loading and unloading curves are linear in the low-force regime; however, they are characterized by different slopes. The loading and unloading curves are linked by an almost flat characteristic. To investigate the behavior in more detail and to understand its origin, we present additional measurements in Figure 2. Figure 2a shows the results obtained by repeating the force–deflection measurements for several cycles on the same micropillar. It can be observed that the pattern is independent of the number of applied cycles and no permanent deformation of the micropillar or micropillar substrate contact occurs. The observation demonstrates the stable clamped condition of the micropillar base to the PEN substrate. If the base was broken by the mismatch of mechanical properties between the micropillar and the substrate, different cycles should show different curves due to the non-elastic process during acquisitions. Similarly, Figure 2b shows results obtained by varying the speed of the AFM probe movement. Also in this case, a very good agreement between cycles with different speeds is found, thus excluding that viscous effects play a relevant role in the mechanical response of the micropillar. Finally, we performed cyclic tests with increasing force amplitudes, as reported in Figure 2c. It is found that the area enclosed between the loading and unloading branches increases linearly with the applied force. A similar behavior has been reported and modeled by Pratt et al. in the case of cantilever-on-cantilever AFM measurements.^49^ The effect was attributed to friction between the AFM tip and the bent beam and also applies to our experimental conditions. Due to the friction, a force component builds up during loading that is oriented normal to the tip axis. As a consequence, a bending moment results at the free end of the AFM cantilever, causing its rotation. Such additional rotation leads to a modification of the laser position on the PSPD, which results in a misleading modification of the measured force.

**Figure 3:**
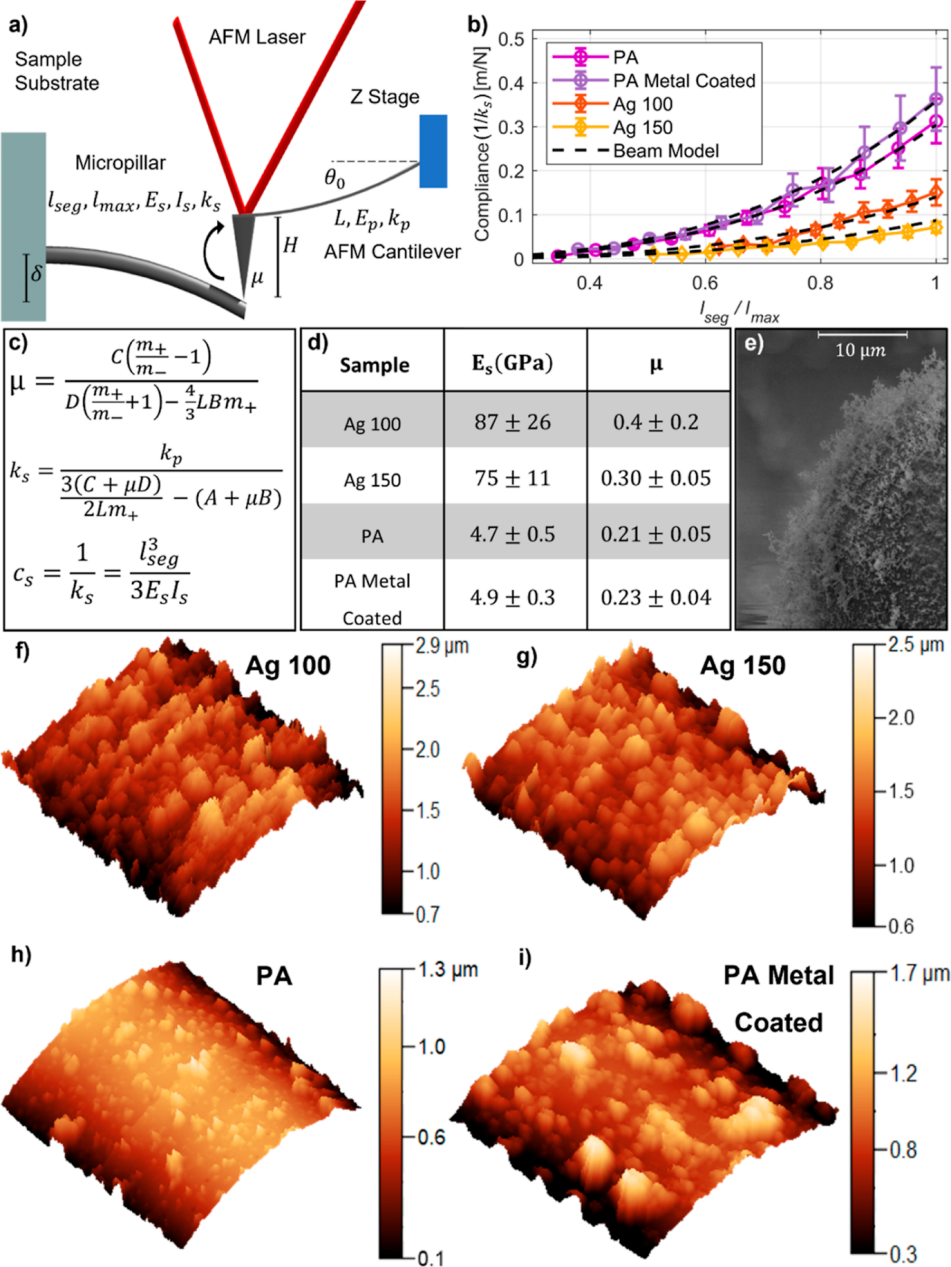
Interpretation of micropillar force–deflection curves: (a) Scheme reports the AFM cantilever bending a micropillar showing the main parameters of the model explaining hysteresis. (b) Graph shows the measured compliance (1/*k*_s_) as a function of the position of the AFM probe on the micropillar. (c) Main formulas of the model. (d) Elastic modulus and friction coefficient values obtained for PA, metal-coated PA, and Ag with two different sintering temperatures (100 and 150 °C). (e) SEM image of a metal-coated PA micropillar surface. (f–i) AFM images (10 × 10 μm^2^) of the side surfaces of the micropillar of different materials.

To compensate for this effect, a correction strategy is developed based on an analytical model. The sketch reported in Figure 3a shows the sample and the AFM tip during a force spectroscopy measurement, together with the main variables of the analytical model. The model assumes that the additional bending moment due to friction is influenced not only by the cantilever tilt (θ_0_), the coefficient of friction (μ), and the ratio between the tip height and the cantilever length (*H*/*L*) but also by the stiffness of the sample (*k*_s_). The sample stiffness is here defined as the proportionality factor existing between a concentrated force applied in a predetermined point along the micropillar height and the corresponding displacement measured at the same point. In the model, AFM cantilever is assumed to be characterized by stiffness (*k*_p_). The normal force *F* is linearly related to the frictional force calculated as μ*F*. It is now worth mentioning that in AFM experiments, forces are estimated by measuring the variation of the cantilever free-end rotation (θ) while moving the tip along the *z*-axis (*Z*). Usually, the conversion from the angle θ to the force amplitude is done automatically by the instrument according to beam theory. Therefore, applying the reverse conversion to the force values provides the angle θ needed. In particular, the converting formula is given by: . Under the assumptions introduced above, as detailed in,^49^ the slope of the *θ*(*Z*) curves can be calculated as
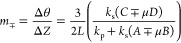
1where the minus sign corresponds to the loading curve, while the plus sign corresponds to the unloading curve. The dimensionless parameters *A*, *B*, *C*, and *D* depend on the tilt angle θ_0_ and, in particular, *A* = cos^2^ θ_0_ – 3*H*/2*L* cos θ_0_ sin θ_0_, *B* = cos θ_0_ sin θ_0_ + 3*H*/2*L* cos^2^ θ_0_, *C* = cos θ_0_ – 2*H*/*L* sin θ_0_, *D* sin θ_0_ + 2*H*/*L* cos θ_0_.

When the slopes of the loading and unloading curves are known, eq 1 provides a system of two equations and two unknowns, which can be used to determine the friction coefficient μ and the micropillar stiffness *k*_s_. In particular
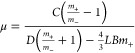
2
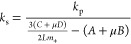
3

Once the stiffness of the sample is correctly determined, its dependence on the distance from the micropillar base can be studied. To do this, we acquired five force spectroscopies every 50 μm along the micropillar starting from the tip to mid-height. The results are shown in Figure 3b. Instead of stiffness, we report the compliance (), as it allows for a better fit procedure of the measurements obtained at a larger distance from the micropillar’s base. The data show an increase in compliance along the micropillar height following a cubic relationship. Such a power-law is in agreement with the mechanical model of a beam with a constant circular cross-section, according to the Euler–Bernoulli beam model. Therefore, the relationship between micropillar length and compliance is given by^48,50^
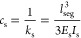
4where *I*_s_ is the moment of inertia of the section, *E*_s_ is the elastic modulus of the sample. In the case of a circular cross-section of radius *r*, the moment of inertia is given by . *l*_seg_ is the distance from the base of the micropillar to the point of contact between the tip and the specimen. *l*_seg,_ thus, corresponds to the effective length of the specimen. Using eq 4, we fit the experimental data acquired at different positions to estimate the elastic moduli. The obtained fits are reported as dashed lines in Figure 3b and show excellent agreement with the measurement data. We note that the elastic modulus obtained in this way describes the global behavior of the micropillar and does not provide information on local variations in elastic properties, as would be accessible by nanoindentation experiments.

To understand the reproducibility of our measurements, we analyzed, for each sample type, two 3D inkjet-printed micropillar arrays for a total of 16 micropillars. The model fits all the experimental data well. The table in Figure 3d shows the average values of elastic moduli and friction coefficients, with standard deviations reported as measurement uncertainties. Our results show that the values of elastic moduli of micropillars are comparable with the corresponding bulk values (76 GPa for Ag and 2–4 GPa for PA ink after curing).^51,52^ Furthermore, we note that different sintering temperatures do not result in a noticeable change in the elastic moduli of the silver micropillars. In fact, the measured values for the microfabricated samples at 100 and 150 °C are comparable considering the uncertainties. The large variability of elastic moduli is related to intrinsic variability from one micropillar to another, as caused by the printing-based microfabrication strategy. Additionally, it must be noticed that eq 4 depends on the micropillar length cubed. Therefore, small errors in the micropillar length result in a considerable variation of the elastic moduli. Nevertheless, these findings highlight that it is much more effective to change the micropillar length or diameter rather than the sintering temperature to tune its stiffness for the proper application. Also, the PA and metal-coated PA samples have comparable elastic moduli. Therefore, we note that the metal coating has no significant impact on the stiffness of the PA sample. This is related to the fact that the thickness of the metal coating is only 150 nm, while the overall diameter of the  μm. It is therefore possible to microfabricate conductive micropillars from nonconductive inks without increasing their stiffness. Figure 3e–i show images of the morphology of different micropillars obtained by SEM and AFM in the non-contact mode over areas of 10 μm^2^. The images show that the surfaces of PA and metal-coated PA micropillars are visually smoother than those of Ag. This is confirmed by the friction coefficient estimated from the fit, which is lower for the case of coated and uncoated PA samples than for the Ag ones. It must be noticed that metal coating, composed of 10 nm of Ni and 150 nm of Pt, increases the roughness of the sample with respect to the uncoated samples; this is also highlighted by a slightly higher friction coefficient.

## 3 Conclusions

This paper reports the characterization of the elastic and frictional properties of 3D inkjet-printed micropillars fabricated with different inks and post-treatment procedures. The characterization method relies on AFM experiments that measure micropillar bending and forces at different micropillar segment lengths. To analyze the resulting stiffness data and to extract the relevant material and surface properties, an analytical mechanical model is provided. The AFM method is easy to conduct, is not destructive, and does not require particular sample preparation. Accordingly, the experiments are simpler than typical micromechanical experiments performed inside a scanning electron microscope. To correctly apply the method, two aspects must be accounted for. First, it is necessary to ensure that the compliance of the micropillar substrate is sufficiently small. This has been overcome by thermally bonding the substrate to a glass carrier. Second, it is necessary to ensure that the AFM laser is not blocked by the sample itself before reaching the PSPD. This can be easily obtained by positioning the micropillars at the edge of the sample.

The investigated micropillars were printed with different inks and parameters. One kind of sample was based on silver nanoparticles sintered at 100 and 150 °C. A second kind of sample was PA micropillars with and without a metallic coating. We decided to test Ag micropillars, as they have already been used for in vitro applications.^9^ We then tested PA micropillars, as the material has an elastic modulus lower by almost 2 orders of magnitude, confirming that the experimental method can also be used for less rigid materials. Our experimental findings show that all micropillars can be modeled as beams with a constant circular cross-section. The elastic moduli determined for the silver samples prepared at different sintering temperatures are comparable to each other, suggesting that this does not play a major role in the mechanical properties of the micropillars. Similarly, for the case of PA samples, the coating does not provide a measurable alteration of the mechanical properties, but it changes the roughness of the micropillar surfaces. Although a significant number of micropillars were measured, relatively small standard deviations were obtained, demonstrating the reproducibility of our method.

In conclusion, such measurements provide access to the mechanical properties of 3D inkjet-printed micropillars employing easy-to-access laboratory instrumentation and well-established AFM techniques. The method allows for rapid estimation of mechanical properties, thus giving the possibility to parametrize the microfabrication steps and investigate their impact on the final device repeatedly. This paves the way for tuning the mechanical properties of 3D-printed micropillars on demand for different applications.

## 4 Experimental Section/Methods

### 4.1 3D Inkjet-Printing Micropillar Arrays

Three-dimensional, printed micropillar arrays were printed on a 125 μm thick PEN substrate with an inkjet printer (CeraPrinter F-Series, Ceradrop) using 1 pL cartridges (DMC-11601, Fujifilm Dimatix). Two different inks were used: silver nanoparticle (Silverjet DGP 40LT-15C, Sigma-Aldrich) and UV-curable PA ink (DM-IN-7003-I, Dycotec Materials Ltd).

#### 4.1.1 Silver 3D Inkjet-Printed Micropillars

Following the procedure described in the literature,^9^ Ag micropillars were printed with 3212 droplets. Samples were thermally sintered at 100 and 150 °C for 2 h. After sintering, the samples were cooled back down to room temperature in 1 h. Using a three-axis UV laser marker (MD-U1000C, Keyence) the substrates were cut to the desired dimensions (2.5 × 1 cm). The laser used a shutter frequency of 100 kHz set at 1.5 kW and a writing speed of 100 mm s^–1^. The outline was etched with the laser with a total of 100 repetitions.

#### 4.1.2 PA 3D Inkjet-Printed Micropillars

Prior to printing, the PA ink was allowed to equilibrate to room temperature before being filtered through a 0.22 μm polyethersulfone filter (TPP) and loaded into a cartridge, which was covered with Al foil to protect the content against light. For the UV-curable PA ink, the same waveform as previously described was used.^9^ The nozzle plate and the sample stage were held at 40 and 50 °C, respectively. With an appropriate working distance, 400 droplets of PA ink were ejected to form micropillars. In order to form the 3D shape, individual droplets were consecutively cured (1 J cm^–2^) layer-by-layer.

#### 4.1.3 Metal-Coated PA 3D Inkjet-Printed Micropillars

PA 3D structures were coated with Ti (10 nm, deposition rate of 0.1 nm/s), followed by Pt (150 nm, deposition rate of 0.2 nm/s), using a high vacuum-coating system (BAL-TEC Med 020, LabMakelaar Benelux BV). The pressure inside the deposition chamber was 7.2 μbar.

### 4.2 3D-Printed Micropillars’ Length and Diameter Measurements

The lengths of micropillars range from 700 to 1100 μm. The length values have been obtained as the difference between the *Z* positions of the AFM probe when it has been approached to the top and to the base of the 3D-printed micropillars, respectively. The average diameter of Ag micropillars is 33 ± 1 μm, while for  ± 1 μm. The diameters have been measured by exploiting the optical microscope mounted on top of the AFM probe.

### 4.3 Sample Preparation for AFM Mechanical Characterization

SU8 3005 epoxy resin (KAYAKU, Advanced Materials) was used to bond the 3D-printed micropillar array printed on PEN foils to glass slides. The dimensions of the slides were 1 cm in width, 2.5 cm in length, and 1 mm in thickness. The resin was continuously spin coated using three different speeds. An initial speed of 500 rpm was used for 10 s (*a* = 100 rpm/s), followed by 3000 rpm for 30 s (*a* = 100 rpm/s), and finishing with 6000 rpm for 10 s (*a* = 500 rpm/s) in order to avoid edge effects.^53^ The resin was pre-cured by soft baking at 95 °C for 3 min (hotplate, Harry Gestigkeit -3T) with the foil containing the micropillars on top. For curing, an OtoFlash (model G171, NK-Optik GmbH) system with 2000 flashes set at a wavelength of 365 nm was used. Finally, the samples were again heated on the hot plate using three different temperatures. An initial temperature of 65 °C (1 min) was used, followed by 95 °C (3 min), and finishing with 150 °C (2 min). The temperature variations were done gradually (3 min). Once cooled to room temperature, the PEN film is bonded to the carrier glass slide. To allow AFM characterization, 3D inkjet-printed micropillars were positioned close to the carrier substrate border (ca. 150 μm).

### 4.4 AFM Tip Calibration

The AFM system used in this work is NX 10 from Park System with an AFM tip 25Pt300B from Rocky Mountain Nanotechnology. The sensitivity of the tip (19.5 ± 0.3 V/μm) was calibrated on a silicon surface. The force constant for the AFM cantilever (18 N/m) was provided by the manufacturer. To avoid possible indentation effects of the AFM tip in the micropillar, we used a tip with a radius of curvature of 1.5 μm. The radius was obtained by starting from the value given by the manufacturer (10 nm) and scratching the tip on a Si sample. The radius was measured by performing several indentations on a polydimethylsiloxane sample of known elastic modulus (*E* = 2 MPa) and fitting the curves applying the Hertz’s model for the case of a spherical rigid indenter.

## Supporting Information Available

SEM images of the internal structure of micropillars, and investigation about the indentation of the AFM probe into the micropillar during the bending experiment (PDF)
