# Supplementary material for: Determination of Stiffness and the Elastic Modulus of 3D-Printed Micropillars with Atomic Force Microscopy–Force Spectroscopy
Source: ACS Appl Mater Interfaces. 2023 Jan 27;15(5):7602–9. doi: 10.1021/acsami.2c21921 (PMC9923676; doi:10.1021/acsami.2c21921)
Supplement: Supplementary file 1 — am2c21921_si_001.pdf [file am2c21921_si_001.pdf]

Supporting Information

Determination of Stiffness and the Elastic Modulus of 3D-printed micropillars with Atomic Force Microscopy-Force Spectroscopy

Giorgio Cortelli [a], Leroy Grob [c], Luca Patruno [a], Tobias Cramer [b], Dirk Mayer [d], Beatrice Fraboni [b], Bernhard Wolfrum [c], Stefano de Miranda [a]*

[a] *Department of Civil, Chemical, Environmental and Materials Engineering*
University of Bologna
Viale del Risorgimento 2, 40136, Bologna, Italy

[b] *Department of Physics and Astronomy*
University of Bologna
Viale Berti Pichat 6/2, 40127, Bologna, Italy

[c] *Neuroelectronics, Munich Institute of Biomedical Engineering, Department of Electrical Engineering*
Technical University of Munich
85748 Garching, Germany

[d] *Institute of Biological Information Processing (IBI-3)*
Forschungszentrum Jülich GmbH
52425 Jülich, Germany.

Corresponding Author
Tobias Cramer
E-mail: tobias.cramer@unibo.it

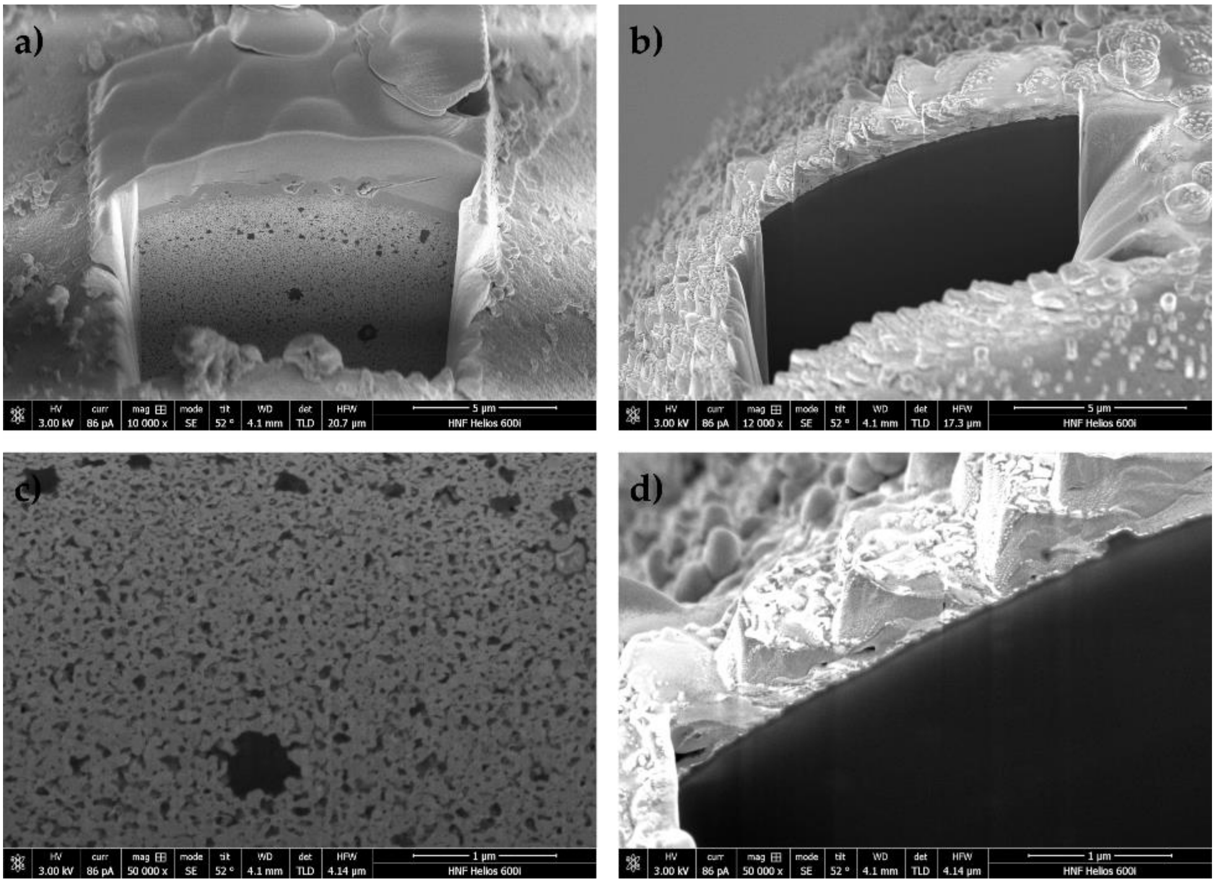

Figure S1. Scanning electron microscope images of focused ion beam cut a), c) 150°C sintered silver nanoparticle based and b), d) UV cured polyacrylate based micropillar. All images show the internal structure after bulk milling and polishing.

While bending a micropillar in the configuration shown in **Figure 1b**, there are three possible contributions to the displacement along the Z-axis: AFM cantilever deflection, micropillar deflection, and indentation of the AFM tip into the micropillar surface. The indentation is dependent on the AFM tip radius of curvature, the force applied, and the indented material. Well-established models such as the Hertz model[1] and the Oliver – Pharr model[2], describe the relationship between the material parameters and the indenter parameters. The Hertz model is usually used to investigate soft materials, while the Oliver – Pharr model is applied to data acquired on stiff materials. Our investigations on the indentation of the AFM probe into the micropillars highlight that in our case we don’t need to apply any contact mechanics model to keep into account the indentation, as the indentation is negligible compared to the micropillar bending. To determine the amount of indentation, we acquired force spectroscopies on the micropillar in a vertical configuration, to avoid any deflection of the micropillar. **Figure S2a** shows a sketch of the experimental setup. A typical force indentation curve acquired in this

configuration on a PA micropillar is reported in **Figure S2b**. The graph shows that the indentation is smaller than a few nanometers, and it can be considered negligible.

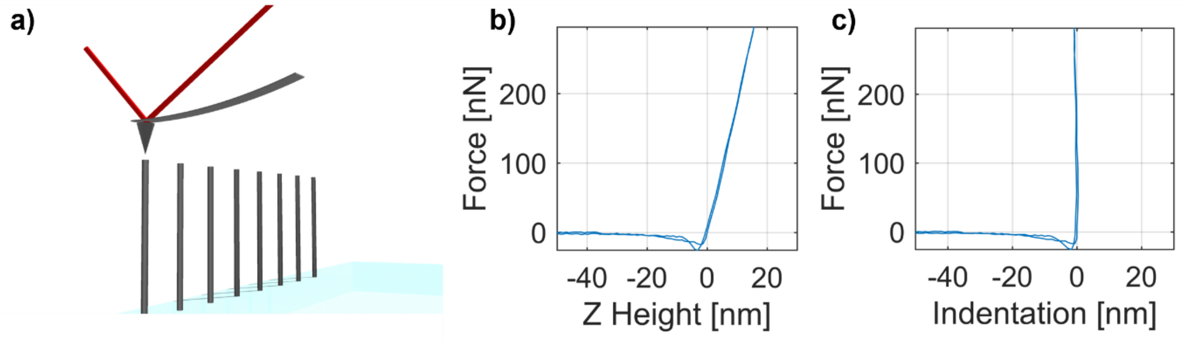

Figure S2. a) Scheme of the experimental setup used to estimate the indentation of the AFM tip inside the micropillar surface. b) Typical force-displacement (Z-height) curve obtained estimating the indentation on a PA micropillar. c) Typical force-indentation curve obtained subtracting in the x-axis the AFM cantilever deflection from the force-displacement plot. Note that the indentation is close to zero.

References

(1) Hertz, H. On the Contact of Elastic Solids. *Z. Reine Angew. Math.* **1881**, *92*, 156–171.

(2) Oliver, W. C.; Pharr, G. M. An Improved Technique for Determining Hardness and Elastic Modulus Using Load and Displacement Sensing Indentation Experiments. *J. Mater. Res.* **1992**, *7* (6), 1564–1583. https://doi.org/10.1557/jmr.1992.1564.
